# Cholesterol Granuloma in the Maxillary Sinus: A Rare Presentation Associated With an Odontogenic Cyst

**DOI:** 10.7759/cureus.43041

**Published:** 2023-08-06

**Authors:** Ahmed Z Abdelkarim, Ahmed Fereir, Ahmed M Elzayat, Scott Lozanoff, Sushil Paudyal

**Affiliations:** 1 Division of Oral and Maxillofacial Radiology, Ohio State University, Columbus, USA; 2 Department of Oral and Maxillofacial, Faculty of Dentistry, Future University in Egypt, Cairo, EGY; 3 Department of Oral and Maxillofacial Surgery, Insurance Hospital, Cairo, EGY; 4 Department of Anatomy, Biochemistry & Physiology, University of Hawaii School of Medicine, Honolulu, USA; 5 Department of Internal Medicine, Quaid e Azam Medical College, Bahawalpur, PAK

**Keywords:** dentistry, nasal polyps, odontogenic cysts, maxillary sinus, cholesterol granuloma

## Abstract

Cholesterol granuloma is a histopathological finding characterized by a mass of connective tissue and granulation tissue. It is primarily observed in the middle ear, mastoid process, or paranasal sinuses, with rare occurrences in the dental odontogenic region. A dentigerous cyst encloses the crown of an unerupted tooth by expanding its follicle and attaches to the neck of the tooth. Here, we report a 63-year-old female who presented to the dental clinic complaining of an ill-fitted denture. A panoramic radiograph showed a well-defined radiolucent lesion in the upper left maxillary sinus with an impacted third molar. Computed tomography revealed loss of the anterior and lateral sinus walls. The cyst was enucleated surgically. The final diagnosis was confirmed by histopathological examination, which revealed focal areas of cholesterol clefts in the cystic wall of the dentigerous cyst.

## Introduction

Cholesterol granuloma (CG) is a histopathological entity with a cystic appearance [1]. It is identified by the occurrence of fibrous granulation tissue filled with cholesterol crystals surrounded by macrophages containing hemosiderin and giant cells [2]. The most common sites for the occurrence of CG are the middle ear (especially in the cases associated with chronic middle ear diseases) and mastoid; however, it has also been reported in the brain, lungs, kidneys, breast, and testis. It was 1984 when the first published paper in the English literature by Kaffe et al. introduced the term CG for a better description of a cystic mass in the palate associated with odontome [3].

Dentigerous cysts are odontogenic cysts that develop from a tooth follicle around the crown of an impacted or unerupted tooth. It is the most common developmental cyst, accounting for up to 20% of all cysts of the jaws lined by epithelium [4]. When inflamed, dentigerous cyst walls occasionally contain cholesterol crystals, hemosiderin pigments, and Rushton’s hyaline bodies, similar to those reported in radicular cysts [5].

Very few cases of CG of the jaw associated with the wall of dentigerous cysts have been reported to date [4,6,7]. Herein, we report the radiographic and histopathological features of a case of CG of the maxillary sinus that occurred in a dentigerous cyst.

## Case presentation

**Figure 1:**
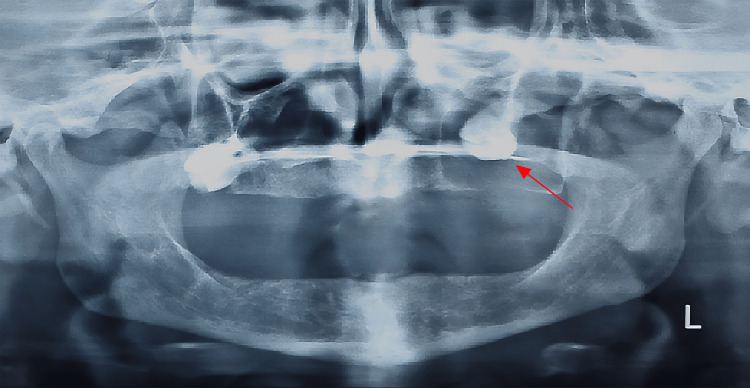
Panoramic radiograph examination. An impacted upper left third molar into the left maxillary sinus (red arrow) with bone resorption and complete pneumatization of the sinus inferiorly till the alveolar crest level was noticed.

**Figure 2:**
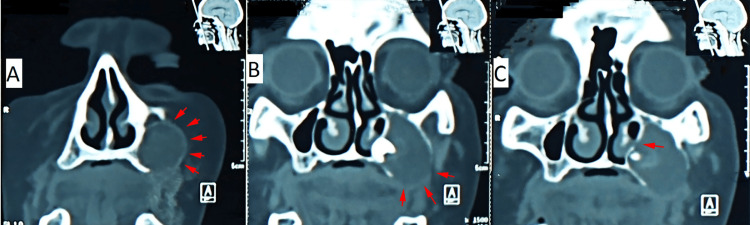
Multi-slice computed tomography (coronal sections). A: Large hypodense, expansile, well-demarcated, and osteolytic mass extending from the maxilla into the maxillary sinus (red arrows). B: An impacted third molar was abnormally displaced toward the lateral wall of the nose and the left inferior conchae with loss of the alveolar bone border and part of the lateral sinus wall (red arrows). C: Loss of the integrity of the medial wall of the sinus was noticed in a coronal section (red arrows).

A 63-year-old female patient was referred to the Department of Oral and Maxillofacial Surgery with swelling on the left side of the face and a chief complaint of a recent ill-fitted denture. The patient reported that the problem started after she noticed a slowly growing bulge in her upper jaw three months before. Upon intra-oral examination, a swelling ranging from firm consistency to eggshell cracking was noticed at the left upper posterior maxillary side and occluding the buccal vestibule. There was no fistula, and the swelling was asymptomatic with no signs of local infection. A panoramic radiograph revealed an impacted upper left third molar into the left maxillary sinus with bone resorption and complete pneumatization of the sinus inferiorly until the alveolar crest level (Figure 1). Multi-slice computed tomography (MSCT) revealed a large hypodense, expansile, well-demarcated, osteolytic mass extending from the maxilla into the maxillary sinus (Figure 2A). An impacted third molar was abnormally displaced toward the lateral wall of the nose and left inferior conchae, with loss of the alveolar bone border and part of the lateral wall of the sinus (Figure 2B). Loss of integrity of the medial wall of the sinus was observed in the coronal section (Figure 2C). Owing to the size of the lesion and its association with the impacted tooth, a differential diagnosis of a dentigerous cyst, keratocyst odontogenic tumor, and ameloblastoma was considered.

**Figure 3:**
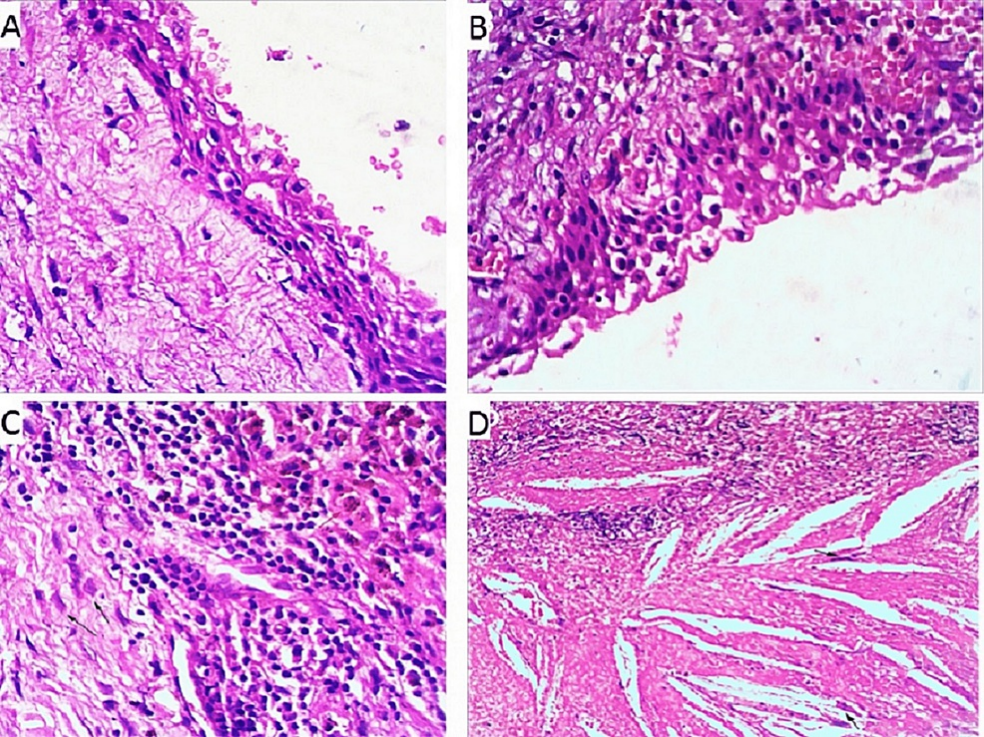
Histopathological examination. A: The cystic wall focally lined by pseudostratified columnar and stratified non-keratinizing squamous epithelium. B: Areas of focal ulceration, hemorrhage, and secondary infection. C: Acute and chronic inflammatory cells heavily infiltrated the cyst wall with Multinucleated foreign-body giant cells, lymphocytes, ghost cells, and a few hemosiderin-laden macrophages. D: A large collection of longitudinal clefts, which were indicative of cholesterol crystals surrounded by foreign body giant cells and macrophages filled with hemosiderin.

The lesion was surgically approached through the anterior maxillary wall under general anesthesia using the Caldwell-Luc approach. Complete enucleation of the lesion with the wisdom tooth was performed. The opened cystic mass measured 4.5 × 3.5 × 1.5 cm with a gray smooth inner lining. Microscopic examination revealed a cyst wall focally lined by pseudostratified columnar and stratified non-keratinizing squamous epithelium (Figure 3A) with areas of focal ulceration, hemorrhage, and secondary infection (Figure 3B). Acute and chronic inflammatory cells heavily infiltrated the cyst wall with multinucleated foreign-body giant cells, lymphocytes, ghost cells, and a few hemosiderin-laden macrophages (Figure 3C). A large collection of longitudinal clefts, indicative of cholesterol crystals surrounded by foreign-body giant cells and macrophages filled with hemosiderin (Figure 3D). No evidence of cytological atypia, malignancy, keratinization, or ameloblastic formation.

## Discussion

Here, we present a rare case of CG in the wall of a dentigerous cyst associated with an impacted molar and involving the maxillary sinus. To the best of our knowledge, there are 12 published articles, and a comprehensive review of the scientific literature on this topic was conducted, encompassing relevant publications from 1984 to the present [1-4,6-13]. Furthermore, aside from our reported case, instances of CG in the oral cavity have been documented to either arise from an odontogenic source or extend from the maxillary sinus, presenting as an isolated intra-oral swelling (Table 1). In addition to the above-mentioned articles, only one case report published in 1975 by Wickenhauser et al. about intra-oral CG was published in German [14]. 

**Table 1:** Cases diagnosed with cholesterol granuloma in the oral cavity. Odontogenic origin or extension from maxillary sinus.

No.	Year	No. of cases	Author	Age	Gender	Site
1	2014	2	Alkan et al. [1]	A) 57	A) Male	A) Maxilla
				B) 67	B) Female	B) Maxilla
2	2017	1	Fernández-Olarte et al. [3]	31	Female	Mandible
3	1984	1	kaffe et al. [5]	11	Male	Maxilla
4	2016	3	Kamboj et al. [6]	A) 45	A) Male	A) Maxilla
				B) 38	B) Female	B) Mandible
				C) 47	C) Male	C) Mandible
5	2010	1	Lee et al. [8]	68	Male	Mandible
6	2012	1	Bhullar et al. [9]	43	Male	Mandible
7	2018	1	Baldini et al. [10]	46	Male	Maxilla
8	2013	1	Aparna et al. [11]	68	Female	Mandible
9	2014	1	Netto et al. [12]	62	Male	Mandible
10	2017	1	Elo et al. [13]	50	Female	Maxilla
11	2017	2	de Freitas filho et al. [14]	A) 45	A) Female	A) Maxilla
				B) Not mentioned	B) Female	B) Maxilla
12	1988	1	Hirshberg et al. [15]	55	Female	Mandible

It seems that CG could be formed in any type of odontogenic cyst as a reaction to cholesterol crystals [9,15]. Cholesterol crystals are more commonly found in inflammatory cysts, especially in radicular cysts. In contrast, CG has been reported to demonstrate a relatively low incidence of developmental cysts, including odontogenic keratocysts [7]. In fact, all reported cases of CG that originated in the maxilla were related to the maxillary sinus, except for one case, which was related to an odontome in the palate [3].

The patient’s main complaint was a recent ill-fitted denture due to intra-oral swelling. Despite the invasion of this mass into the maxillary sinus, the patient did not report any history of rhinorrhea, sinusitis, facial pain, or nasal obstruction, which are the usual nonspecific symptoms similar to cases reported with invasion into the maxillary sinus [16].

This opens the door to a dilemma regarding the pathogenesis of CG. The crystal clear etiology of CG has not yet been established; however, in these cases associated with the maxillary sinus, it could be explained by inadequate drainage or hemorrhage in the sinus following trauma with the consequences of hemolysis in the sinus, as cholesterol may come from the cell membrane of the erythrocytes. Along with impaired drainage, this cholesterol could be precipitated as crystals, which later evokes an inflammatory response as a foreign body with subsequent migration of leukocytes and macrophages and the formation of giant cells [17,18].

Yamazaki et al. [19] introduced the first published article on CG at the molecular level, which suggested that perlecan, which is present in the cystic wall of immature granulation tissue, could be related to the formation of cholesterol granulomas.

In the present case, the lesion was part of an odontogenic cyst that involved the maxillary sinus. Both of these factors could be attributed to etiological factors. Almada et al. [20] pointed out the possibility of development of the CG from primary or secondary odontogenic origin and invading nearby structures such as maxillary sinuses, as close anatomical structures. DeFreitas Filho et al. echoed this in 2017 [12], who reported two cases of CG in a cystic mass in the maxillary sinus after dental treatment.

The potential conditions to consider include odontogenic keratocyst, mandibular hemangioma, aneurysmal bone cyst, mandibular AVM (arteriovenous malformation), central giant cell granuloma, Ewing sarcoma, juvenile ossifying fibroma, osteosarcoma, or Brown's tumor.

There is no specific radiographic presentation for CG, and it could occur in both jaws with or without odontogenic cysts [1,4], as the diagnosis made will be based on histopathological examination. However, the Treatment of the CG in the oral cavity is mainly surgical excision, usually by enucleation, and recurrence is quite rare [2].

## Conclusions

CG found in the wall of a dentigerous cyst associated with an impacted molar and involving the maxillary sinus is not considered a distinct clinical condition but rather a nonspecific histopathological reaction to cholesterol crystals. As its clinical and radiographic characteristics are nonspecific, the findings of this study emphasize the significance of considering CG in the differential diagnosis of odontogenic cysts and tumors. Histopathological analysis is essential for a correct final diagnosis of CG.

Recognition of the true nature of the entity as a dentigerous cyst obviated the need for a different line of surgical treatment such as hemi-maxillectomy. We agree with Baldini et al., who performed systematic surgical enucleation as soon as the diagnosis of such lesions was made in order to avoid any complications related to its growth. We need more studies on CG cases in the oral cavity to learn more about their unique characteristics and better understand this rare condition.
